# Biochemical Engineering Perspective on cGAS: From Enzyme Discovery to Potential Industrial Application

**DOI:** 10.1002/cbic.202500942

**Published:** 2026-02-25

**Authors:** Makram Fataeri, Katrin Rosenthal

**Affiliations:** ^1^ Faculty of Technology Bioprocess Engineering Bielefeld University Bielefeld Germany

**Keywords:** biocatalysis, bioprocess, cGAMP, cGAS, cyclic dinucleotides

## Abstract

Since its discovery as a pivotal enzyme in innate immunity, cyclic GMP‐AMP synthase (cGAS) has been extensively studied for its immunological significance and catalytic mechanism. However, its potential as a biocatalyst for the efficient synthesis of the second messenger 2′3′‐cyclic GMP‐AMP (2′3′‐cGAMP) remains underexplored. This review provides a comprehensive biotechnological perspective on cGAS, highlighting its enzymatic and structural features, substrate promiscuity, homologs, and engineered variants. We examined the expression systems reported in previous studies and assessed their suitability for scalable cGAS production. Furthermore, we explored reaction engineering strategies for 2′3′‐cGAMP synthesis by comparing published production and purification methods. This review aims to bridge the gap between fundamental enzymology and applied bioprocessing by positioning cGAS as a promising biocatalyst for the pharmaceutical industry, with potential applications in immunotherapy, vaccine adjuvants, and beyond.

## Graphical abstract

This review examines the structural and enzymatic properties of cyclic GMP‐AMP synthase, including substrate promiscuity, homologs, and engineered variants. In addition, it evaluates potential expression systems for large‐scale enzyme production and analyzes reaction and purification strategies for the synthesis of cyclic dinucleotides to connect fundamental enzymology to bioprocess development for pharmaceutical manufacturing.

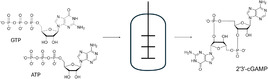

## 1 Introduction

The discovery of the cyclic GMP‐AMP synthase (cGAS)‐stimulator of interferon genes (STING) pathway in 2013 marked a major milestone in our understanding of the innate immune response. Prior to this discovery, it was already known that cytosolic DNA triggers the production of type 1 interferons via the STING pathway. The identification of cGAS as a cytosolic DNA sensor completed the signaling pathway from DNA detection to cytokine production [1].

**SCHEME 1:**
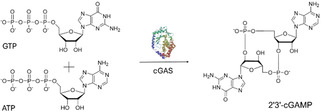
Represention of the cyclization of ATP and GTP to 2′3′‐cGAMP catalyzed by the enzyme cGAS.

When double‐stranded DNA (dsDNA) is present in the cytosol of the cell, whether as a result of infection or due to damaged nucleus or mitochondria, cGAS (EC 2.7.7.86) acts as a DNA sensor by binding to the DNA and undergoing a conformational change [2, 3]. This structural rearrangement activates the enzymatic function of cGAS, enabling it to catalyze the synthesis of the cyclic dinucleotide (CDN) 2′3′‐cyclic GMP‐AMP (2′3′‐cGAMP) from ATP and GTP. This reaction is represented in Scheme 1.

Acting as a second messenger, 2′3′‐cGAMP binds to the STING receptor located on the endoplasmic reticulum, which activates the cytosolic kinases IKK (I kappa B kinase, EC 2.7.11.10) and TBK1 (TANK binding kinase 1, EC 2.7.11.1) and thus their corresponding transcription factors NF‐κB and IRF3 [2]. These factors cooperatively induce the expression of type 1 interferons and proinflammatory cytokines, thereby regulating the innate immune response [2]. Given its central role in linking cytosolic DNA sensing to immune response activation, the cGAS‐STING pathway, and particularly 2′3′‐cGAMP, has gained significant interest in the pharmaceutical industry especially in cancer biology and autoimmune disorders [4, 5, 6], and more recently in sepsis‐associated organ dysfunction and age‐related neurodegenerative disorders [7, 8]. As a result, researchers focused on the chemical synthesis of 2′3′‐cGAMP and on optimizing its stability, cellular uptake, and therapeutic efficacy. This research led to several derivatives of 2′3′‐cGAMP, such as MK‐1454, a diphosphorothioate with hydroxyl‐to‐fluorine substitutions at the ribose, reaching preclinical and clinical trials [9, 10].

Nowadays, different chemical synthesis pathways are used to produce CDNs with the phosphoramidite synthesis route being one of the most used routes to produce 2′3′‐cGAMP. This method consists of eight steps over several days and results in a low yield of 5% [11, 12]. In contrast, the biocatalytic route using cGAS is a one‐step reaction with an assay yield that can reach up to 95% within 24 h on a milliliter scale [13, 14]. So far, the biocatalytic reaction has only been demonstrated on a milliliter scale, although theoretical studies have already shown that it is significantly more environmentally sustainable than the chemical synthesis route [13]. Very few research was dedicated to the upstream bioprocessing potential of cGAS for the biosynthesis of 2′3′‐cGAMP and its derivatives. Optimized expression systems, tailored protein engineering strategies, robust reaction setup, and downstream processes that ensure high yields and purity should be examined thoroughly for an efficient biotechnological production of 2′3′‐cGAMP.

**FIGURE 1:**
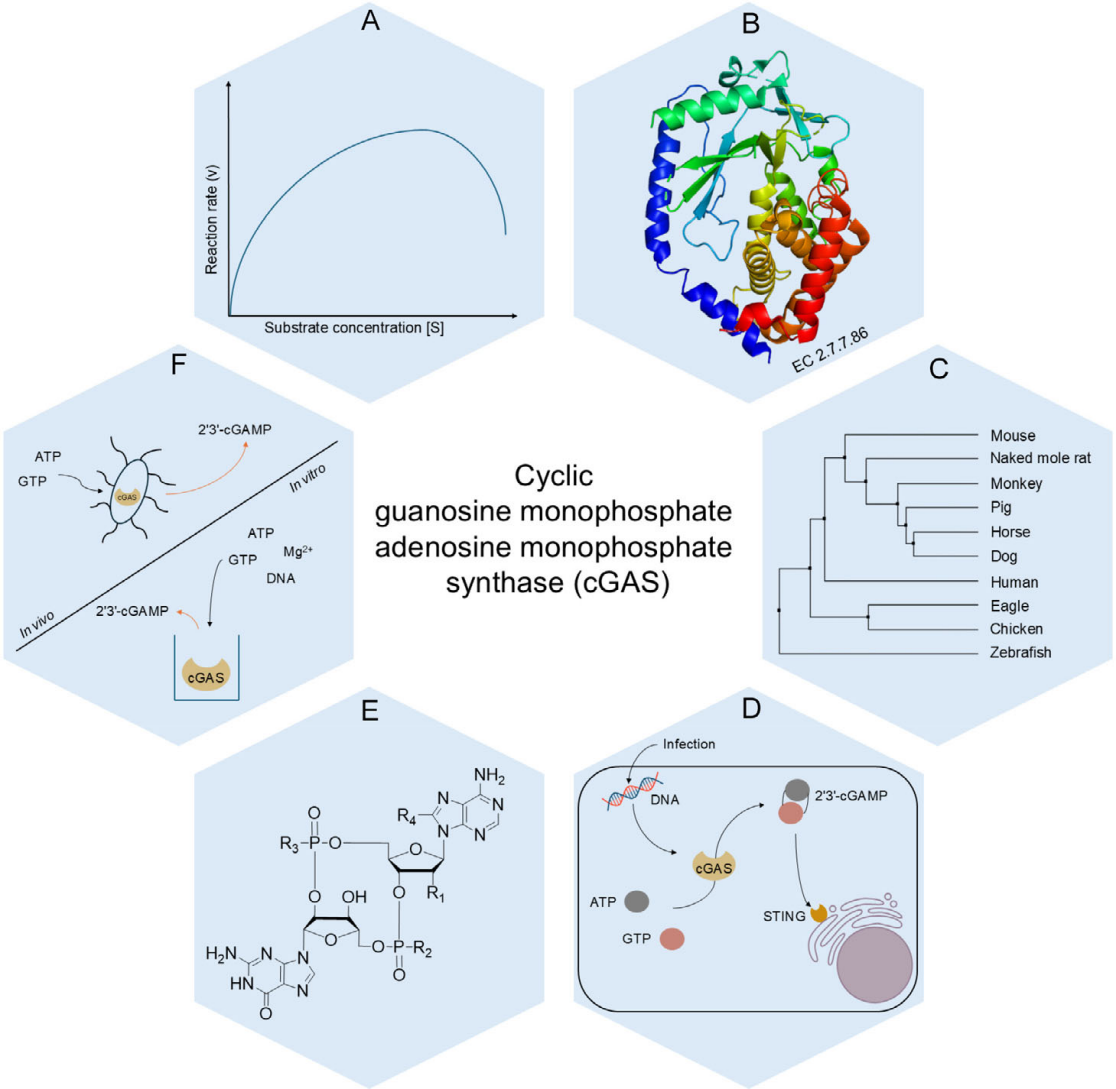
Advances in cGAS (EC 2.7.7.86) research. (A) Michaelis–Menten curve illustrating the enzyme kinetics of cGAS with substrate inhibition. (B) Structure of human cGAS (PDB ID: 4MKP). (C) Phylogenetic analysis of cGAS across representative species. The tree was conducted in Jalview from amino acid sequences to illustrate the evolutionary conservation of cGAS among species. (D) Illustration of cGAS‐STING pathway in a cell. Due to infection or damage of the nucleus or mitochondria, DNA enters the cells and activates cGAS. cGAS can then cyclize ATP and GTP to form 2′3′‐cGAMP. This can then activate the STING receptors on the endoplasmic reticulum, stimulating the innate immune response. (E) Chemical structure of cyclic dinucleotide (CDN) derivatives produced by cGAS or cGAS variants. Shown are 2′3′‐cGAMP (R_1_, OH; R_2_, O^−^; R_3_, O^−^; R_4_, H), 2′3′‐cG^s^A^s^MP (R_1_, OH; R_2_, S^−^; R_3_, S^−^; R_4_, H), and analogs (R_1_, OH/F/Cl/MeS; R_2_, O^−^; R_3_, O^−^; R_4_, H) and (R_1_, OH; R_2_, O^−^; R_3_, O^−^; R_4_, Br/Cl/NH_2_/N_3_). (F) Production of 2′3′‐cGAMP in vitro using an engineered *E. coli* strain to perform the reaction and in vivo using purified enzymes as the biocatalyst.

This review aims to address this gap by covering the key aspects of bioprocess development for 2′3′‐cGAMP synthesis using cGAS, ranging from the biochemical and structural characteristics of cGAS through host selection and protein production to reaction engineering, substrate recycling, and product purification. A schematic overview of the progress made to date is presented in Figure 1.

## 2 Catalytic Mechanism of cGAS

Structural analysis revealed that cGAS has homology with the oligo adenylate synthase 1 (OAS1), which acts as a cytosolic double‐stranded RNA sensor [15], and homology with the *Vibrio cholerae* enzyme dinucleotide cyclase (DncV), which catalyzes the synthesis of 3′3′‐cGAMP [16]. This led to classifying cGAS under the cGAS/DncV‐like nucleotidyltransferase (CD‐NTase) family [17]. cGAS contains a disordered N‐terminal region, which plays a key role in mediating cGAS attachment to chromatin in the nucleus and to the plasma membrane [18, 19]. The C‐terminus, which is responsible for the activity of the enzyme, contains one NTase core domain and one male abnormal 21 homology domain with a zinc thumb motif [20]. cGAS has three DNA binding sites A, B, and C. Site A is responsible for the conformational changes in cGAS, and together with Site B, they facilitate the dimerization formation, whereas Site C strengthens the contact between cGAS and DNA to ensure the phase separation [21].

Upon binding to dsDNA, structural rearrangements of the active site are induced to enable the synthesis of 2′3′‐cGAMP. The cGAS–dsDNA interactions lead to the formation of dimers (2:2 DNA‐cGAS dimeric molecules) and liquid–liquid phase separation (LLPS) [22, 23]. The DNA‐bound cGAS can then catalyze the cyclization of ATP and GTP in two steps. In the first step, the 2′‐OH of GTP, positioned within the active site of the enzyme, attacks the α‐phosphate of ATP, after its activation by the cofactor Mg^2+^, resulting in the formation of a 2′5′‐linked linear dinucleotide (pppGpA). In the second step, the α‐phosphate of the GTP unit of the linear intermediate is attacked by the 3′‐OH of ATP unit to form 2′3′‐cGAMP [3, 10].

Studying the distribution of CD‐NTases across different species revealed the widespread occurrence of these enzymes, spanning from bacteria to vertebrates. These findings led to the identification of various cGAS homologs across different species. In addition to the well‐described human and mouse cGAS, studies also focused on porcine, dog, monkey, and chicken cGAS [24, 25, 26]. Moreover, in 2020, four new homologs were identified: Przewalski's horse, naked mole‐rat, bald eagle, and zebrafish [27]. On the other hand, screening the bacterial CD‐NTases showed that different homologs can produce different dinucleotides like 3′3′‐cUAMP, which is produced by *Escherichia coli* CdnE, and 3′3′‐cGAMP, which is produced by DncV [17]. However, the discovery of bacterial 2′3′‐cGAMP only occurred in 2023, when Tak et al. identified enzymes from *Bacillus thuringiensis* (BtCdnB) and *Clostridium botulinum* (CbCdnB) capable of producing 2′3′‐cGAMP to activate the bacterial pathway equivalent to cGAS‐STING pathway called cyclic oligonucleotide‐based antiphage signaling systems [28].

Kinetic studies of cGAS show a sigmoidal curve when the catalytic rate is plotted against substrate concentrations [29, 30]. A surface plasmon resonance assay was used to determine the kinetic constants for 2′3′‐cGAMP formation. The *K*_M,ATP_ reached a value of 190 µM and the *K*_M,GTP_ a value of 90 µM, while the maximum catalytic rate (*k*_cat_) for the truncated human cGAS was determined to be 5 min^−1^. In another study, the mouse cGAS showed values of 86 µM for *K*_M,ATP_ and 96 µM for *K*_M,GTP_ with a *k*_cat_ of 23 min^−1^ [31]. Moreover, competitive substrate inhibition was detected for human cGAS with a *K*_IS,ATP_ of 2500 µM in a GTP concentration‐dependent manner. Notably, when a constant molar ratio of ATP and GTP (up to 2 mM) was used, no inhibition was detected [29]. Hooy et al. tested the effects of dsDNA length, metal cofactors, and ion concentrations on the enzymatic activity. They found out that longer dsDNA (339 bp) increases the *k*_cat_ when compared to shorter dsDNA (19–72 bp). They also showed that the catalytic activity can be increased by two‐ to fourfold when a mixture of Mn^2+^ and Mg^2+^ is added [32]. Another study by Du et al. showed that Zn^2+^ can also enhance the activity of cGAS by enhancing the phase separation with DNA, which emphasizes the importance of LLPS on the activity of cGAS [33]. A study in 2024 linked the importance of phase separation to limiting the activity of exonuclease TREX1 in the cytosol, which is responsible for the degradation of cytosolic DNA [34]. In addition to the effect of ions on LLPS, it was observed that various viral and human proteins exerted a comparable influence on LLPS and, consequently, enzymatic activity [35]. Examples of these proteins are the human Ras‐GTPase‐activating protein SH3 domain‐binding protein 1 (G3BP1), which enhance cGAS activity; the RNA‐binding protein ZCCHC3 which, when bound to cGAS, exhibits a greater affinity for DNA than cGAS alone; and the Herpes simplex virus type 1 VP22 (HSV1‐VP22), which inhibits the cGAS activity by excluding cGAS from the condensed phase [36, 37, 38]. Recently, Tang et al. introduced two dimerization systems to alternate the phase separation: light‐inducible pMag‐nMagHigh1 dimerization system and chemical‐inducible FRB‐FKBP dimerization system. These systems are also applicable to other accessory proteins such as ZCCHC3 and PQBP1 [39].

Beyond modulating the phase separation, recent efforts have focused on enhancing the interaction of 2′3′‐cGAMP with the STING receptor. The affinity of 2′3′‐cGAMP toward the STING receptor can be increased through the incorporation of phosphothioate diester linkages. In 2014, Li et al. chemically synthesized a bisphosphothioate analog of 2′3′‐cGAMP (2′3′‐cG^s^A^s^MP) that showed improved properties in immuno‐oncology therapy because of its ability to escape the hydrolysis activity of the enzyme ENPP1 [40]. This led to the investigation of cGAS abilities to produce other unnatural 2′3′‐cGAMP analogs with enhanced STING stimulation [14, 25]. Studies have shown that human cGAS exhibits substrate promiscuity, where substrates with unnatural nucleotides aside from the natural ATP and GTP produced new CDNs. Different 2′3′‐cGAMP analogs were produced by using different substrates with modifications at the nucleobase, ribose, and α‐thio phosphate.

## 3 Biocatalytic Production of 2′3′‐cGAMP

In recent years, research has been conducted on structural and biochemical characterization of cGAS. However, the knowledge utilizing the enzyme as a biocatalyst for the production of 2′3′‐cGAMP is still limited. In the following, we will consider advances relating to the use of cGAS as a biocatalyst, including expression systems, enzyme and reaction engineering, and downstream process.

### 3.1 cGAS Expression Systems

The efficient production of 2′3′‐cGAMP relies on the ability of the host cell to produce active cGAS at a scale. For an economical process, it is necessary that sufficient quantities of the functional enzyme can be produced. Different studies showed that cGAS can be heterologously produced in prokaryotic and eukaryotic host systems. *E. coli* is one of the most widely used host systems for recombinant protein production, because of its rapid growth, low cultivation costs, ease of genetic manipulation, and being one of the best studied model organisms [41]. Among *E. coli* strains, *E. coli* BL21 (DE3) is the most commonly used strain for cGAS production, because of the higher protein stability achieved due to the absence of the proteases Lon and OmpT [27, 41]. In some cases, *E. coli* Rosetta (DE3) has been used to improve the translation of rare codons [39, 41, 42]. In addition, a study showed that human and mouse cGAS can be also synthesized in *Salmonella typhimurium* in an attempt to activate the STING pathway in vitro [43]. Despite that, recombinant production of proteins in prokaryotes comes with some challenges, including cellular toxicity and lack of eukaryotic post‐translational modifications (PTMs), which can lead to protein misfolding, reduced insolubility, or loss of functional activity [44]. In eukaryotic hosts, cellular assays and signaling studies typically employed cGAS expressed in mammalian systems such as HEK293T cells, while other investigations used cGAS produced in High Five insect cells [37, 45]. These cells are capable of producing correctly folded and functional proteins due to the natural eukaryotic PTMs (with some limitation in case of insect cells), but they usually have more complex growth condition, require expensive media and equipment, and result in lower yields [46].

In a study published in 2020, cGAS was produced in an *E. coli*‐based cell‐free protein synthesis system [27]. This system can be used for a high‐throughput screening for the expressibility of genes, where the coding gene template is added to a mixture of amino acids, nucleotides, ions, energy regeneration components, and macromolecular crowding agents. This results in a quick and cheaper screening method. The disadvantage, however, lies in its limited scalability [27, 47].

In order to facilitate the solubility of the recombinant protein and the purification process, it is possible to utilize a variety of different fusion proteins. In case of cGAS, the most widely employed tag to enhance solubility and structural stability is small ubiquitin‐related modifier (SUMO) [27]. Alternatively, some studies have tagged cGAS with the glutathione S‐transferase (GST), which functions as a solubility enhancer and affinity tag [48, 49]. In this context, GST can bind to immobilized glutathione, facilitating purification processes. Maltose‐binding protein was also used as a fusion protein to increase the solubility of cGAS and for purification via single‐step affinity to cross‐linked amylose [30]. In most studies, a polyhis tag is used for the purification of cGAS, whereby histidine residues (initially in sixfold form, with the possibility of additional attachment) are added to the N‐terminus of the SUMO tag. Immobilized metal affinity chromatography can then be used to purify HIS‐tagged cGAS, in which the negatively charged histidine can interact with metal ions such as Ni^2+^, Co^2+^, Cu^2+^, and Zn^2+^ bound to a matrix [27, 49]. During scale‐up, purification would likely be replaced by nontag‐based methods such as ion exchange chromatography, or alternatively, the tag could be used for immobilization, thereby significantly simplifying purification [50]. Different strategies have been described by Barbosa et al. on how to couple immobilization and purification of proteins [51]. These techniques are already being used today by some of the biggest companies, such as BASF and Evonik, on an industrial scale in food, chemical, and pharmaceutical industries [52].

### 3.2 Engineering of cGAS

In recent years, there has been a significant focus among researchers on substituting different amino acids in cGAS [53]. This approach aims to enhance our understanding of the enzyme and potentially expands its functional capabilities. A range of approaches can be identified in literature. Some of these approaches focus on targeting the DNA‐binding interface, while others involve introducing mutations to the catalytic core or altering the nucleosome binding ability. Moreover, several studies were conducted that focused on the phase separation domains or zinc fingers [21, 53]. In 2022, a study was published about the biocatalytic synthesis of MK‐1454 from two thiotriphosphate nucleotides [9, 54]. For that, an engineered cGAS variant was developed to cyclize the non‐natural substrates in a stereocontrolled transformation. Through a series of seven cycles of directed evolution, the activity of the bald eagle cGAS was enhanced by several orders of magnitude. These mutations were designed with a focus on enabling cGAS to cyclize non‐natural substrates using non‐natural cofactors to produce MK‐1454 and with a focus on improving substrate tolerance and enzyme stability. Another research group focused on stabilizing the activated form of cGAS employing a multi‐state computational protein design framework [55]. They successfully engineered constitutively active cGAS variants using this approach. These variants function independently of dsDNA binding, oligomerization, or phase separation [55]

Aside from the amino acid mutations, certain studies have concentrated on the PTMs of cGAS [24]. Serine and threonine phosphorylation has been observed to inhibit cGAS activity, while tyrosine phosphorylation has been shown to enhance enzyme activity [56, 57, 58, 59, 60]. Furthermore, ubiquitin ligases were found to influence cGAS, as demonstrated by the testing of several ubiquitin ligases. An exemplification of this phenomenon is the E3 ligase, which, through the process of monoubiquitation, enhances dimerization, DNA binding, and enzyme activity [61, 62, 63]. Research findings demonstrated that the deubiquitination of K48‐linked ubiquitin led to the stabilization of cGAS [64, 65]. This phenomenon has also been observed in the context of SUMOylation of cGAS [66]. Furthermore, it has been demonstrated that acetylation has the capacity to either activate or deactivate cGAS, a process that is dependent upon the specific acetylation site [67, 68, 69].

Beyond that, no studies have reported engineered cGAS with improved activity or stability, and no quantitative data describing these properties are available in the literature.

### 3.3 Reaction Engineering for 2′3′‐cGAMP Production

To advance from mechanistic insight toward industrial application, it is essential to consider how reaction engineering can optimize substrate use and reactor design. To date, a variety of production modes have been employed for small‐scale production of 2′3′‐cGAMP [27, 70, 71]. In some publications, whole cells were used as biocatalysts, while in others, purified enzymes were used for the in vitro production of 2′3′‐cGAMP. These purified enzymes were also tested in two production approaches: a single‐step reaction with ATP and GTP as substrates and a multi‐step reaction involving multiple free enzymes in an enzyme cascade. Additionally, immobilization could theoretically be applied to cGAS to enhance its stability and reusability.

In a study published in 2024, a titer of 186 mg/L 2′3′‐cGAMP was produced and secreted into the medium by using a whole‐cell biocatalyst, *E. coli* BL21(DE3), and heterologously produced mouse cGAS [72]. On the other hand, using purified mouse cGAS for in vitro production of 2′3′‐cGAMP from ATP and GTP resulted in 274 mg/L [27]. To reduce the substrate costs, the in vitro 2′3′‐cGAMP production can be started from an earlier stage, for instance, from adenosine and guanosine. In principle, the synthesis of 2′3′‐cGAMP could be coupled to various nucleotide triphosphate generating enzyme cascades, including systems based on acetate kinases, pyruvate kinases, creatine kinases, or even electrochemistry‐coupled synthesis [73, 74, 75, 76, 77]. To date, however, not many of these nucleotide triphosphate synthesis cascades have been coupled to 2′3′‐cGAMP synthesis. Successful examples include cascades combining nucleoside kinases with polyphosphate kinases or adenylate/guanylate kinases with an acetate kinase [9, 70]. The highest reported 2′3′‐cGAMP titer achieved using such an enzyme cascade was 971 mg/L, obtained with a combination of nucleoside kinases, polyphosphate kinases, and cGAS [78].

**FIGURE 2:**
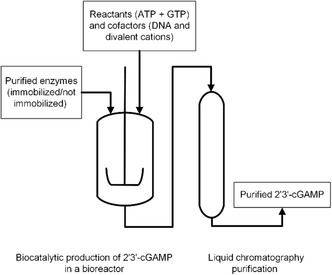
Potential bioprocess for the production of 2′3′‐cGAMP.

With respect to reactor design, all studies to date have been conducted in batch mode in either shaking flasks or reaction tubes [72, 78]. These initial studies prove the feasibility of recombinant cGAS in producing 2′3′‐cGAMP. However, they do not yet address reactor choice for upscaling. An overview of a potential, generalized bioprocess for 2′3′‐cGAMP production can be found in Figure 2. This consists of two operation units, one unit for the upstream where either free or immobilized enzymes can be mixed with reactants and cofactors in batch, fed‐batch, or continuous stirred tank reactor (STR) followed by a chromatographic purification step of the product.

A considerable challenge in the process of upscaling arises from the use of isolated cGAS, particularly in relation to substrate inhibition. What remains unclear is whether the enzyme is product‐inhibited, which to our knowledge has not yet been tested. One potential solution for substrate inhibition would be the utilization of fed‐batch reactors, where substrates are added gradually to the bioreactor via feed lines as a function of time [79]. This enables the prevention of substrate accumulation, thereby avoiding the inhibition of the biocatalytic reaction. As for using whole‐cell biocatalysts, fed‐batch reactors are the preferred option for industrial‐scale applications, since some nutrients or substrates above a certain concentration can cause inhibition [80]. Consequently, cultivating in a fed‐batch reactor results in a higher cell density (>50 g_CDW_/L) compared to that achieved in a batch reactor (5–10 g_CDW_/L). Furthermore, metabolic by‐products that exhibit inhibitory effects can be avoided by utilizing fed‐batch reactors [80]. Another option for producing 2′3′‐cGAMP on a larger scale is to use a plug flow reactor, particularly a packed‐bed reactor (PBR) with immobilized enzymes, such as cGAS or the other enzymes involved in the cascade. PBRs are widely used in industrial applications due to their continuous mode, high yields, and ability to recycle enzymes [81, 82].

Altogether, these reaction engineering approaches highlight the critical role of process design in enabling the efficient and scalable production of 2′3′‐cGAMP, paving the way for future industrial applications of cGAS as biocatalyst.

### 3.4 Purification of 2′3′‐cGAMP

Aside from establishing strategies for 2′3′‐cGAMP synthesis, the downstream process plays a crucial role in ensuring product purity and maintaining bioactivity. To date, however, little research has focused on optimizing this stage. Table 1 shows the production titers achieved until now to produce 2′3′‐cGAMP during upstream and downstream processes. The most commonly reported methods for 2′3′‐cGAMP purification in the literature are anion exchange chromatography (AEC) and reverse‐phase high‐performance liquid chromatography (RP‐HPLC) [14, 83]. Kulkarni et al. purified 60 mg/L 2′3′‐cGAMP via AEC achieving a product recovery of 32% with endotoxin levels below 0.3 EU/µg [72]. Similarly, a recovery yield of 33% was achieved using RP‐HPLC purification [14].

**TABLE 1:** Titers of 2′3′‐cGAMP achieved with today's upstream and downstream methods.

2′3′‐cGAMP production		Titer (mg/L)	References
Upstream	Whole‐cell biocatalysis	186	[72]
	Free enzymes	274	[14]
	Enzyme cascade	971	[78]
**2′3′‐cGAMP purification**			
Downstream	Anion exchange chromatography (AEC)	60	[72]
	RP‐HPLC	90	[14]

In 2019, Lv et al. described a purification method for CDNs comprising three major steps: (1) purification using STING immobilized affinity resin, (2) solid‐phase extraction with a microporous adsorption resin (SP207), and (3) final purification with C18 ODS‐AQ column. This approach achieved a concentration exceeding 82 mg/L and purity greater than 99%, which corresponds to over 53% recovery [71].

Another purification method for 2′3′‐cGAMP was identified in the literature, involving liquid–liquid extraction with a mixture of phenol, chloroform, and isoamyl alcohol in a ratio of 25:24:1 [84]. To date, however, no further published studies have employed this method. In contrast, in a large‐scale production of another CDN, c‐di‐GMP, a combination of AEC and solvent precipitation was used to purify the CDN [85]. The phosphoramidite‐based chemical synthesis of c‐di‐GMP, first reported in 2010, achieved efficient purification with a 10‐min crystallization step using acetone with an overall yield of 17%−19%. With the addition of a single AEC step, this method has since become the commonly adopted method for the chemical production of 2′3′‐cGAMP [13, 11, 86, 12].

## 4 Challenges and Outlook

Although the enzymatic synthesis of 2′3′‐cGAMP via cGAS has shown promising results on laboratory scale, numerous challenges persist before the process can be scaled up for industrial application. At the enzyme level, cGAS exhibits a low turnover rate and a low specific activity. It also requires DNA and other metal cofactors for activation. Furthermore, the limited stability of cGAS poses significant challenges for scaling up production and for maintaining its efficacy over extended periods. This might also be the reason why 100% substrate conversion has not yet been achieved. At the processes level, substrate inhibition and the high cost of substrates represent major bottlenecks that limit overall efficiency and scalability, while upscaling chromatography steps presents financial and technical challenges. Furthermore, the polar, hydrophilic nature of 2′3′‐cGAMP, combined with its low titers in a water‐based reaction buffer, poses a significant challenge for product recovery.

For an environmental and economic assessment of a process in early development stages, different metrics should be considered. In 2022, Meissner et al. suggested four metrics for a better evaluation of the development requirements. These are reaction yield (*Y*_reaction_), biocatalyst yield (*Y*_biocatalyst_), product titer, and space‐time yield (STY) [87, 88]. Table 2 summarizes the benchmark values for these process metrics and the biocatalytic 2′3′‐cGAMP production values as of today.

**TABLE 2:** Benchmark values and current process metrics for 2′3′‐cGAMP production via cGAS.

	Benchmark values [88]	Collected data	
		Whole‐cell biocatalysts [72]	Free enzymes [14]
*Y*_reaction_ [%]	>90	—	87
*Y*_biocatalyst_ [*g*_product_/*g*_biocatalyst_]	50–500	0.16 g/g_CDW_ a	6.86
Product titer [*g*_product_/*L*_reactor_]	10–50	0.19	0.27
STY [*g*_product_/*L*_reactor_·h]	1–10	0.01	0.07

a Assuming that an OD_600_ of 1 is equivalent to 0.3 g/L of cell dry weight [89].

As shown in Table 2, the biocatalytic production of 2′3′‐cGAMP using free enzymes achieves higher values than those achieved using whole‐cell biocatalysts. For example, the STY was 7‐fold higher and the product titer was 1.4‐fold higher when free enzymes were used compared to whole cells [14, 72]. All numbers, however, show that the biocatalytic production of 2′3′‐cGAMP is far from being commercially viable. These metrics help to set targets for the process development because they serve as indirect indicators for the economy of the process. For example, the reactor cost can be estimated from the STY values, while the contribution of the biocatalyst to the total process cost can be derived from the *Y*_biocatalyst_ [90]. After defining the target performance metrics, biocatalyst and process optimization can be pursued to achieve the desired outcome. Several approaches may be considered, for instance, protein engineering to improve cGAS stability and activity. Mclntosh et al. have demonstrated this by engineering bald eagle cGAS to produce MK‐1454 [9]. Additionally, immobilization could extend the enzyme's lifetime and enable reusability. This technique is already used in various biocatalytic processes. To reduce the financial burden, multi‐enzyme cascades that regenerate nucleotides from inexpensive precursors may be considered, while fed‐batch biocatalytic reactions could help overcome substrate inhibition, as described in Section 3.3. Together, these approaches provide a versatile toolbox for advancing cGAS biocatalysis from proof‐of‐concept studies toward scalable and economically viable applications.

## 5 Conclusion

Overall, research on CDNs has expanded rapidly during the last decade, demonstrating their importance. However, advancing cGAS as biocatalyst toward industrial application requires further development in enzyme engineering, bioprocess engineering, and purification strategies. Despite the fact that cGAS may not yet be ready for industrial application, because of its limited stability, susceptibility to substrate inhibition, and the lack of upscaling strategies, further research into this system is promising. The enzyme could enable scalable synthesis of CDNs, opening new possibilities for pharmaceutical applications such as immunotherapy and vaccine adjuvants.

## Funding

This study was supported by Bundesministerium für Wirtschaft und Klimaschutz (KK5681701AJ4).

## Conflicts of Interest

The authors declare no conflicts of interest.
